# Genome-Wide Polygenic Risk Score for Predicting High Risk Glaucoma Individuals of Han Chinese Ancestry

**DOI:** 10.3390/jpm11111169

**Published:** 2021-11-09

**Authors:** Yu-Jer Hsiao, Hao-Kai Chuang, Sheng-Chu Chi, Yung-Yu Wang, Pin-Hsuan Chiang, Pai-Chi Teng, Tung-Mei Kuang, Aliaksandr A. Yarmishyn, Tai-Chi Lin, De-Kuang Hwang, Shih-Jen Chen, Shih-Hwa Chiou, Mei-Ju Chen, Ai-Ru Hsieh, Chih-Chien Hsu

**Affiliations:** 1 Department of Medical Research, Taipei Veterans General Hospital, Taipei 112027, Taiwan; yj1007hsiao@yahoo.com (Y.-J.H.); kevin1985336@gmail.com (H.-K.C.); paichi.teng@gmail.com (P.-C.T.); yarmishyn@gmail.com (A.A.Y.); shchiou@vghtpe.gov.tw (S.-H.C.); 2 School of Medicine, National Yang Ming Chiao Tung University, Taipei 112304, Taiwan; tmkuang@vghtpe.gov.tw (T.-M.K.); taichilin@hotmail.com (T.-C.L.); m95gbk@hotmail.com (D.-K.H.); sjchen96@gmail.com (S.-J.C.); mjchen9069@gmail.com (M.-J.C.); 3 Department of Ophthalmology, Taipei Veterans General Hospital, Taipei 11217, Taiwan; b101100033@tmu.edu.tw; 4 Department of Statistics, Tamkang University, New Taipei 251301, Taiwan; sc18927@gmail.com (Y.-Y.W.); s94120506@gmail.com.tw (P.-H.C.); 5 Institute of Clinical Medicine, National Yang Ming Chiao Tung University, Taipei 11217, Taiwan; 6 Institute of Pharmacology, School of Medicine, National Yang-Ming Chiao Tung University, Taipei 11217, Taiwan; 7 Institute of Food Safety and Health Risk Assessment, National Yang Ming Chiao Tung University, Taipei 112304, Taiwan; 8 Genomic Research Center, Academia Sinica, Taipei 11529, Taiwan

**Keywords:** glaucoma, genome-wide association studies, polygenic risk score, Asian population, biobank, retrospective study

## Abstract

Glaucoma is a progressive and irreversible blindness-causing disease. However, the underlying genetic factors and molecular mechanisms remain poorly understood. Previous genome-wide association studies (GWAS) have made tremendous progress on the SNP-based disease association and characterization. However, most of them were conducted for Europeans. Since differential genetic characteristics among ethnic groups were evident in glaucoma, it is worthwhile to complete its genetic landscape from the larger cohorts of Asian individuals. Here, we present a GWAS based on the Taiwan Biobank. Among 1013 glaucoma patients and 36,562 controls, we identified a total of 138 independent glaucoma-associated SNPs at the significance level of *p* < 1 × 10^−5^. After clumping genetically linked SNPs (LD clumping), 134 independent SNPs with *p* < 10^−4^ were recruited to construct a Polygenic Risk Score (PRS). The model achieved an area under the receiver operating characteristic curve (AUC) of 0.8387 (95% CI = [0.8269–0.8506]), and those within the top PRS quantile had a 45.48-fold increased risk of glaucoma compared with those within the lowest quantile. The PRS model was validated with an independent cohort that achieved an AUC of 0.7283, thereby showing the effectiveness of our polygenic risk score in predicting individuals in the Han Chinese population with higher glaucoma risks.

## 1. Introduction

Glaucoma is the second leading cause of blindness globally [1], but the irreversibility of glaucoma, as opposed to cataract, highlights the importance of diagnosing and treating it as early as possible. Glaucoma is a chronic progressive disease that is commonly characterized by changes in the optic nerve head as well as the corresponding visual field defects [2]. Current treatment methods of glaucoma are limited to lowering the intraocular pressure (IOP) to slow the disease progression at early disease stages, but a majority of glaucoma cases remain undiagnosed until irreversible optic nerve damage occurs [3]. A follow-up study on patients in the United States with treated primary open angle glaucoma (POAG) found a 9% probability of bilateral blindness and 26% probability of unilateral blindness at 20-years’ follow-up [4]. This could be because the initial stages of visual field abnormalities in glaucoma frequently occur in the periphery nasal field rather than the central field, and functional loss mostly only happens as a late symptom in centrally deteriorating visual field cases. Therefore, given that the prognosis for glaucoma depends on the stage at which it is detected, early detection and treatment are the best ways to preserve the retinal ganglion cells and nerve fiber layers. The understanding of genes associated with glaucoma therefore opens new and important avenues for glaucoma treatment.

Genome-wide association studies (GWAS) are observational studies of the genome-wide genetic variation of different individuals aimed to identify any associations with the traits. Such research usually focuses on the association between single nucleotide polymorphisms (SNPs) and major diseases. GWAS and other genetic and genomic studies have accelerated the discovery of genes associated with glaucoma over the past decade. So far, several GWAS studies of glaucoma have identified over 100 risk loci, including mutations in the *CYP1B1* [5,6,7], *OPTN* [8,9], and *PLEKHA7* [10,11] genes. Despite this, neither the normal functions of *MYOC* nor how *MYOC* mutations result in IOP elevation and subsequently glaucoma have been defined. Further genomic studies will help to extend our understanding of the corresponding biological pathways contributing to the pathogenesis of glaucoma. However, many studies have shown that the incidence of glaucoma is geographically and ethnically variable. The incidence of primary congenital glaucoma (PCG) varies from 1:10,000 in the Western population to 1:1250 in inbred populations such as the Gypsy subpopulation of Slovakia [12], whereas the prevalence of POAG is 2.8–8.8% among the African descendants, much higher than among the Caucasians (1.1–2.1%) and Asians (2.6%) [13,14,15]. The discovery of novel genes and their mutations associated with glaucoma with considerations over ethnic differences is hence essential [16,17]. However, presently, most loci were discovered in the populations of the European ancestry [8,12,18,19]. Hence, a larger GWAS cohort focused on people of East Asian ancestry is needed to identify additional risk loci and complete the glaucoma genetic landscape. With increasing genetic testing, early interventions to highlight glaucoma risks at their earliest onset can allow the best precision management strategies to prevent glaucoma-related vision impairment [20,21]. It can also serve as a genetic basis for identifying new therapeutic targets for the associated pathological pathways and molecular events.

Hence, we conducted a large-scale GWAS for glaucoma using the Taiwan Biobank (TWB) 1.0 and TWB 2.0 databases. The TWB database includes whole-genome sequencing data of the Taiwanese population [22]. TWB (https://www.twbiobank.org.tw/new_web/, (accessed on 28 September 2021) is a prospective cohort study of the Taiwanese population, mainly of Han Chinese ancestry, with genomic data divided into either hospital-based or community-based. The community-based biobank covering all the participants of this study includes repeated measurements of a wide range of phenotypes collected from 148,567 individuals (as of August 2021). TWB recruits 30- to 70-year-old participants with no cancer history across 29 recruitment centers in Taiwan [22]. In this study, a total of 96,715 Han Chinese ancestry subjects were involved, and 16,222,535 loci were analyzed. Glaucoma risk loci that have not been previously reported at genome-wide significance levels were identified. Finally, we constructed a glaucoma polygenic risk score (PRS) for risk stratification of glaucoma to predict glaucoma risk in the Taiwanese population retrospectively. The PRS was modeled based on the TWB2.0 and verified with an independent TWB1.0 cohort. We aimed to provide further evidence for the differential genetic architecture of glaucoma between different ethnic populations and provide a unique PRS model for risk stratification in individuals of Han Chinese ancestry.

## 2. Results

### 2.1. Participant Characteristics in TWB 2.0 and TWB 1.0

There were 68,978 TWB2.0 participants and 27,737 TWB1.0 participants, with TWB2.0 being used as a discovery set for glaucoma risk alleles and TWB1.0 as a validation set. Among the discovery set (Table 1), 1013 self-reported glaucoma individuals (defined as cases), constituted 1.47% of the 68,978 TWB2.0 participants. A total of 36,562 individuals without self-reported glaucoma or related comorbidities were considered as controls (for the list of comorbidities, please refer to the Methods section). The total sample size for the discovery set was 37,575, with the case-control ratio of 1:36. On the other hand, in the validation set (TWB1.0), there was a total number of 7082 individuals, consisting of 450 self-reported glaucoma cases and 6632 controls who had neither glaucoma nor glaucoma-related comorbidities.

The basic characteristics of both discovery (TWB2.0) and validation (TWB1.0) sets are shown in Table 1. In the discovery set, gender distributions were similar between the cases and controls, with females predominating.

### 2.2. Glaucoma Risk Loci

**Figure 1:**
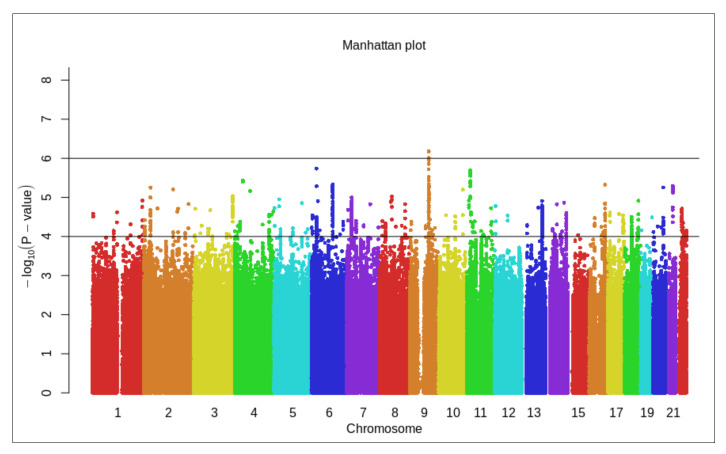
Manhattan plot showing the individual *p*-values against genomic position for individuals (*n* = 1013 cases, *n* = 36,562 controls) from Taiwan Biobank 2.0 (TWB2.0).

After performing adjustments of the Scalable and Accurate Implementation of Generalized mixed model (SAIGE) as described in the Methods section, we conducted a GWAS, and a Manhattan plot for this TWB2.0 study group was generated (Figure 1). Three SNPs had a genome-wide significance level <1 × 10^−6^ while 138 SNPs had *p* < 1 × 10^−^^5^. 

Among the 138 SNPs with *p* < 1 × 10^−5^ (Table S1), variants in 134 SNPs showed positive correlation (OR > 1) while 4 SNPs showed protective effects (OR < 1). The most significantly associated SNPs included positively-associated variants in rs2282199 (G > A, OR = 1.266), rs2282201 (G > A, OR = 1.265), rs10992252 (C > T, OR = 1.261), as well as protective variants in rs903990 (C > T, OR = 0.7992), rs6125932 (A > C, OR = 0.8113), rs62291417 (C > T, OR = 0.6558), and rs145495258 (TTTTCTTTTCTTTTCTTATCG > T, OR = 0.656). Table 2 contains the selected list of SNPs and Table S1 includes the complete list of 138 SNPs. 

### 2.3. Polygenic Risk Score (PRS) and Glaucoma Risk Prediction

**Figure 2:**
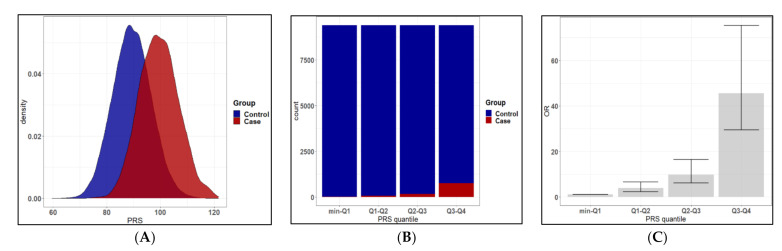
Comparison of glaucoma risks in TWB2.0 classified by PRS_TWB2.0 quantile. (**A**) Distribution of the polygenic risk score (PRS) in glaucoma cases and controls (the red and blue lines represent mean PRS in glaucoma cases and controls, respectively). (**B**) Distribution of cases and controls according to PRS quantiles. (**C**) Odds ratio (OR) for developing glaucoma according to PRS_TWB2.0 quantiles.

To predict glaucoma risk, we constructed a PRS model based on 134 associated SNPs discovered in the TWB2.0 study population. In Table 3, different models based on various combinations of linkage disequilibrium (LD) clumping threshold (r^2^) and genome-wide significance level threshold (p) are listed. The mean PRS was significantly higher in glaucoma cases compared to controls across all models (Table 3 and Figure 2A). After weighing the clinical significance of r^2^, *p*, and AUC, we constructed the model with 134 selected independent SNPs, r^2^ < 0.2, and *p* < 10^−4^ (referred to as PRS_TWB2.0 below) (Table S2).

Among the 134 SNPs, minor alleles in 103 SNPs showed positive correlation (OR > 1) while variants in 31 SNPs showed protective effects (OR < 1). The most significantly associated SNPs included positively-associated variants in rs2282199 (G > A, MAF = 0.311, OR = 1.266), rs4078356 (T > C, MAF = 0.035525, OR = 1.727), rs4757474 (in *PLEKHA7* gene, C > T, MAF = 0.4417, OR = 1.238), as well as protective variants in rs903990 (C > T, MAF = 0.3316, OR = 0.7992 ), rs6125932 (A > C, MAF = 0.46455, OR = 0.8113 ), and rs62468636 (in *STK31* gene, G > A, MAF = 0.40985, OR = 0.8125 ). Table S2 shows the complete list of 134 SNPs.

Regarding the PRS performance, the PRS_TWB2.0 was effective in distinguishing individuals with high glaucoma risks from those with low risks (Figure 2A). The association came up with a dose-response relationship (Figure 2B,C and Table 4). Individuals in the highest quantile of PRS_TWB2.0 (Q3–Q4) had 45.48-fold increased risk compared to the lowest risk quantile (min-Q1), and those in the third (Q2–Q3) and second (Q1–Q2) highest quantile had 9.77 and 3.80-fold risks, respectively. Table 4 shows the case-control distribution among quantiles. Furthermore, in the high-risk group (top 5% to 25% in PRS distribution), Table 5 showed significantly elevated risks of glaucoma: the top 25% of the PRS had a 9.41-fold risk, the top 10% had a 9.72-fold risk, and the top 5% had a 13.30-fold risk of developing glaucoma compared to the remaining population.

**Figure 3:**
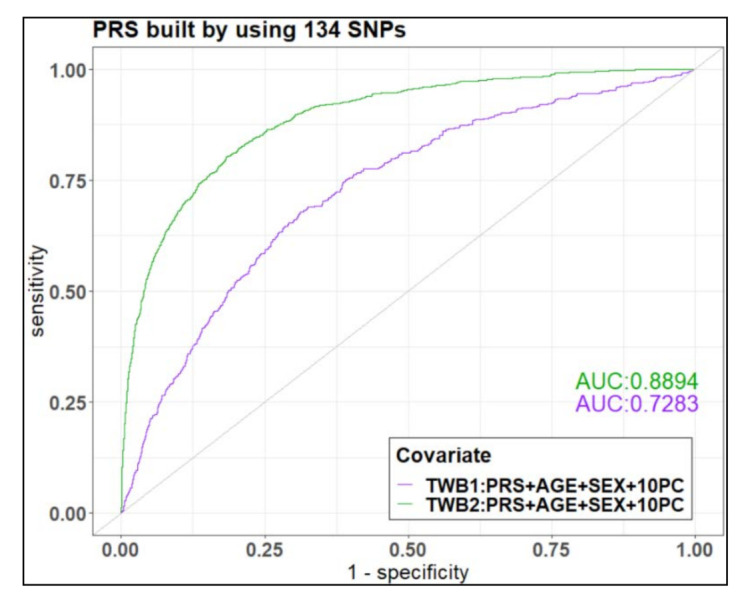
Receiver Operating Characteristic (ROC) curves for the polygenic risk score (PRS) model, PRS_TWB2.0 model, after adjustments for age, sex, and 10 principal components (PCs). The PRS_TWB2.0 model is a PRS model built by 134 SNPs from the TWB2.0 cohort to identify glaucoma cases in the Taiwan Biobank 2.0 (TWB2.0) (green curve) and TWB1.0 (purple curve, for validation) cohorts.

In the PRS_TWB2.0 model, the area under the receiver operating characteristic (ROC) curve was 0.8387 (95% confidence interval = [0.8269–0.8506]). Once additional covariates (age, sex, and first 10 principal components (PCs))) were included in the model, the area under curve (AUC) reached 0.8894 (Figure 3, green curve). As for validation in TWB1.0, we used the PRS_TWB2.0 model adjusted for age and sex with 10 PCs. The corresponding AUC was 0.7283 (Figure 3, purple curve).

## 3. Discussion

The Taiwan Biobank contains high-coverage, whole-genome sequencing data of the Taiwanese population who are mostly of Han Chinese ancestry, a less studied population in glaucoma-related research. In this study, we included 37,575 individuals (1013 cases, 36,562 controls) from TWB2.0 to build a PRS glaucoma prediction model. A total of 138 independent glaucoma-associated SNPs at the significance level of *p* < 1 × 10^−5^ were identified, including the most significant three loci at *p* ≈ 5 × 10^−7^. All except 4 SNPs had an odds ratio larger than 1, indicating greater odds of association with the SNP (exposure) and glaucoma (outcome). Three of these SNPs even displayed odds ratios of >1.9. After LD clumping, 134 selected SNPs were used to construct a PRS that retrospectively predicts glaucoma risk in the Taiwanese population, with an area under the receiver operating characteristic (ROC) curve (AUC) of 0.8387 (95% CI = [0.8269–0.8506]) and 0.8894 after covariates adjustment. Those within the top PRS quantile had a 45.48-fold increased risk of glaucoma compared with those within the lowest quantile. To our knowledge, this is the largest Taiwanese-based PRS glaucoma prediction model to date.

While finding the most supported genes for our 138 newly identified loci is a known challenge, it is nonetheless highly important and relevant for further functional follow-ups. The nearest genes to glaucoma-associated SNPs identified in previous studies using other biobanks include the *CYP1B1* [5,6,7], *OPTN* [8,9], *LTBP2* [23,24], *COL11A1* [25,26], *PLEKHA7* [10,11], *CDKN2B-AS1* [18,27,28], *LOXL1-AS1* [29,30], and *CARD10* [31,32] genes. Among them, only the *PLEKHA7* gene was also found as a supporting gene in our 138 newly identified loci from this Taiwan Biobank population of the Han Chinese ancestry, suggesting marked differences between different ethnicities. By closely analyzing all the nearest genes underlying our 138 newly identified loci, we highlighted the *LINC00475*, *RIPOR2*, and *PLEKHA7* genes to be the most supported genes for the most significant SNPs. Importantly, *PLEKHA7* (Pleckstrin Homology Domain Containing A7) has been associated with Primary Angle Closure Glaucoma. *PLEKHA7* is a gene located at the locus 11p15.2-p15.1 (NCBI Gene ID: 144100) related to the pathway of stabilizing and expanding the E-cadherin adherens junctions. It plays a role of supporting and maintaining adherens junctions through interaction with proteins such as the delta-catenin [33]. PLEKHA7 interacts with CAMSAP3, thereby anchoring microtubules at their minus-ends to zonula adherens and recruiting KIFC3 kinesin to the junctional site [33]. Additionally, PLEKHA7-PDZD11 complex mediates the clustering of ADAM10 at cell–cell junctions with the ADAM10-binding protein TSPAN33 [34]. This mechanism is tightly relevant to the age-related changes in glaucoma. As for the other two genes nearest to the most significant SNPs, *LINC00475* and *RIPOR2*, there has been no reported association to glaucoma and other eye diseases so far. The *LINC00475* is a long intergenic non-protein-coding RNA whereas the *RIPOR2* (RHO Family Interacting Cell Polarization Regulator 2) gene encodes a protein that mediates the polarization of T cells and neutrophils and is a component of hair cell stereocilia essential for hearing.

The list of 138 newly identified SNPs corresponds to 20 unique nearest genes. Three of them, *ROR2*, *SDCCAG8*, and *FXYD6*, are involved in molecular pathways that might have the slightest relation to glaucoma and are of potential interest for further investigations into their potential roles in glaucoma-related mechanisms. *ROR2* (Receptor Tyrosine Kinase Like Orphan Receptor 2) is located on chromosome 9 at location 9q22.31 (NCBI Gene ID: 4920). It encodes the cell surface receptor protein tyrosine kinase and type I transmembrane protein involved in the early formation of chondrocytes, cartilage, and growth plate development. Mutations in this gene can cause brachydactyly type B, the Robinow syndrome, among other skeletal disorders. Besides pluripotency and the GPCR Pathway, ROR2 is also involved in the Wnt signaling pathway where it acts as a receptor for the non-canonical Wnt ligand, WNT5A. A recent study has found that WNT5A was upregulated by dexamethasone (DEX), which in turn regulates aqueous humor outflow by inducing a re-organization of the cytoskeleton in the trabecular meshwork (TM) cells [10]. The knockdown of ROR2 receptor abolished the effects of DEX in TM cells. Given the similarities between DEX-induced glaucoma and primary open angle glaucoma, these results shed light on a potential mechanism to treat primary open-angle glaucoma. *SDCCAG8* (SHH Signaling and Ciliogenesis Regulator) is a gene located on chromosome 1 in the location 1q43–44 (NCBI Gene ID 10806), encoding a protein that organizes the centrosome during interphase and mitosis. It is also involved in epithelial lumen formation, ciliogenesis, and organelle biogenesis. Several studies have also associated the mutations in this gene with retinal-renal ciliopathy. The ciliary localization of SDCCAG8 in photoreceptors is determined by RPGRIP1α, and its absence suppresses their ciliary targeting. However, its association with glaucoma and other ocular diseases remains to be investigated. *FXYD6* (FXYD Domain-Containing Ion Transport Regulator 6) is a gene located on chromosome 11 at the location 11q23.3. It encodes phosphohippolin, a transmembrane protein which is likely involved in the regulation of the activity of Na/K-ATPase. Notably, evidence exists for the therapeutic effects of targeting Na+/K+ ATPases to treat ocular hypertension [12]. The long-lasting effects of decreasing IOP by siRNAs targeting Na+/K+ ATPases is similar to that produced by commercial drugs. Since glaucoma is treated by lowering intraocular pressure, investigation of the involvement of the *FXYD6* gene in glaucoma development may be worthwhile.

The strength of this study lies in its large-scale multi-center approach. Previous studies have reported the differential presentation as well as ethnic and geographic disparities in glaucoma genetics [35,36,37]. We have provided further evidence for the differential genetic architecture of glaucoma between the East Asian and European populations. Further meta-analysis of the UK Biobank and the NHGRI-EBI GWAS Catalog did not improve the genome-wide significance and associations despite after multiple testing corrections. Nonetheless, we replicated and validated our analysis results with the TWB2.0 population with an independent but ethnically similar TWB1.0 population. In general, the SNP effects found in our Taiwanese population are not comparable to genome-wide variants reported in previous studies of other ethnicities and ethnicity remains an important criterion in the genetic basis of glaucoma.

Notably, many of the risk genes we identified yielded a relatively large odds ratio of >1.5. This might be due to the imbalanced case-control data ratio we had, causing statistically significant genes with an odds ratio that has a wide confidence interval to be more easily identified. Therefore, SAIGE association tests were used to further evaluate those genes to account for imbalanced case-control data ratio. However, adopting SAIGE to avoid inflation of type I errors also meant reducing the power of the study [38]. A further limitation of this work is that all the cases of disease status were self-reported, and disease subtypes were not documented. The number of patients with glaucoma was underestimated, and only 1.47% of TWB2.0 participants had glaucoma. According to the Shipai Eye Study in Taiwan, the estimated prevalence of glaucoma, primary open angle glaucoma (POAG), and primary angle-closure glaucoma (PACG) in the elderly population (≥72-year-old) was 8.7%, 3.7%, and 4.8%, respectively [39]. However, the TWB2.0 glaucoma participants are relatively younger, and the prevalence of glaucoma should have been lower. In addition, the proportion of glaucoma types can be different. It may be worthwhile to investigate the association of each of these SNPs with different subtypes of glaucoma.

Furthermore, we also expanded this analysis to derive a PRS that was tested across a wide spectrum of clinically relevant glaucoma outcomes. The significant effect of PRS prediction supports the polygenic nature of the glaucoma-associated risk alleles, i.e., there are many genetic variants with small effects that collectively exert a significant impact on the risk of glaucoma in carriers of the mutations. We also observed similar results for the unweighted PRSs. The inclusion of PRSs resulted in significantly improved prediction accuracy for these homogenous groups compared with the traditional risk factors, showing the clinical utility of biomarkers-based GWAS. Glaucoma is an increasingly significant public health issue given the increasing number of individuals that will be affected by the disease over the next several decades. At present, since glaucoma is irreversible and there is no definitive cure, early detection is crucial for early treatment, and identifying individuals at a greater risk of the disease can further reduce its impact. To estimate the cumulative measure of genetic risk identified through genetic association studies, PRSs aggregate individual variants to better measure of the genetic predisposition for a polygenic health outcome like glaucoma. As a result, early detection of glaucoma can provide high risk asymptomatic individuals (with a higher PRS) with the opportunity for earlier intervention.

## 4. Materials and Methods

### 4.1. Study Population and Genome-Wide Association Study (GWAS)

The participants and their data were obtained exclusively from the TWB (https://www.twbiobank.org.tw/test_en, (accessed on 28 September 2021). Up to 15 April 2021, more than 144,000 participants have been recruited. The demographic and health-related survey data for 105,388 study subjects were released in December 2019. Detailed genotyping and imputation procedures are described by Wei et al. [22]. In brief, 105,388 demographic and health-related survey data points were released in December 2019. There were 95,252 participants who had been genotyped with custom TWB1.0 array (TWB1.0 = 27,737) or TWB2.0 array (TWB2.0 = 68,978).

Control samples with the following comorbidities were removed: asthma, cardiac arrhythmias hyperlipidemia, hypertension, stroke, diabetes, peptic ulcers, depression, epilepsy, migraine, dementia, renal failure, and headache. Sample quality control was carried out to exclude samples with genotyping rates <95%, heterozygosity, and cryptic relatedness. Markers were excluded based on the following criteria: (i) with missing call rate >5%, (ii) with minor allele frequency (MAF) <1%, or (iii) significantly deviated from the Hardy–Weinberg equilibrium (*p* < 1.0 × 10^−6^) using PLINK (v1.9) [40].

After performing quality control for the samples, 11,785,052 variants from 7082 (450 cases, 6632 controls) TWB1.0 and 11,110,260 variants from 37,575 (1013 cases, 36,562 controls) TWB2.0 participants were used in the subsequent analysis. Adjusting sex and age as a covariate, logistic regression case/control analysis was performed using the PLINK software. We performed GWAS in two stages. Firstly, PLINK was used to screen out significant signals for all traits. The genome-wide significance threshold was set at a loose level of *p* = 5.0 × 10^−3^. Secondly, significant signals from the first stage were further selected for association tests using R’s SAIGE package (version 0.35) in order to adjust for the imbalanced case-control ratio.

### 4.2. Polygenic Risk Score (PRS) Analyses

To build the PRS prediction models, we used the standard clumping and thresholding (C + T) method. The hyperparameters for this method were the cut-off of correlation r2 and *p*-value threshold p. The parameter spaces for r^2^ and *p* were {0.2, 0.4, 0.6, 0.8} and {10^−4^, 10^−5^} respectively. For each combination of (r^2^, *p*), we used PLINK with window size 10 Mb to select SNPs. For model selection, we considered TWB2.0 as the training sample report the prediction performance (AUC) and TWB1.0 as the testing sample to evaluate AUC of the prediction model. For the SNPs whose minor alleles showed protective effects on glaucoma, we converted their minor alleles to major alleles as risk alleles, which results in positive weight values for all variants. The PRS analyses were performed using PLINK 1.9. In this study, the predictive abilities of TWB1.0 and TWB2.0 PRS were compared using the area under the receiver operating characteristic (ROC) curve (AUC) [41]. Improvement in AUC between ROC curves were tested using Delong’s method [42]. The analyses were performed using the R package “pROC”.

### 4.3. Statistical Analyses

Continuous data are presented as means with standard deviation, and categorical data are presented as proportions. Our study used Student’s T-test to compare the mean values of continuous variables and chi-squared tests to compare the frequencies of categorical variables between two groups. All statistical analyses were performed using R version 4.1.1.

## 5. Conclusions

In our study, we analyzed data from TWB1.0 and TWB2.0. A total of 138 independent glaucoma-associated SNPs were identified from the TWB2.0 population at the significance level of *p* < 1 × 10^−5^. Among these SNPs, the SNPs rs2282199, rs4078356, and rs4757474 had odds ratios of 1.266, 1.77, and 1.238 and were nearest to the *LINC00475*, *RIPOR2*, and *PLEKHA7* genes, respectively. Further analyses are warranted to survey the risk loci differences between different glaucoma subtypes. Furthermore, we built a novel PRS model to identify patients susceptible to glaucoma, which was validated in an independent cohort from the TWB1.0 database. Further meta-analysis of the UK Biobank and NHGRI-EBI GWAS Catalog did not improve the genome-wide significance and associations, indicating the differential genetic architecture of glaucoma between different ethnic populations. Together with the newly identified experimentally significant loci, the PRS model we have constructed highlights the potential for far-reaching clinical utility in reducing the global burden of glaucoma.

## Figures and Tables

**Table 1:** Characteristics of participants from Taiwan Biobank 2.0 (TWB 2.0) and 1.0 (TWB 1.0).

	Discovery TWB2.0 (*n* = 37,575)			Validation TWB1.0 (*n* = 7082)			Statistics and *p*-Values ^1,2^
Variables	Case (*n* = 1013)	Control (*n* = 36,562)	*p*-Value ^1^	Case (*n* = 450)	Control (*n* = 6632)	*p*-Value ^1^	
Sex							
Male(%)	360 (35.54)	10,889 (29.78)	9.196 × 10^−5^	225 (50)	3116 (47)	0.2335	<2.2 × 10^−16^
Female(%)	653 (64.46)	25,673 (70.22)		225 (50)	3516 (53)		
Age(years)	58.18 ± 0.576	47.7 ± 0.108	<2.2 × 10^−16^	58.4 ± 0.4786	50.06 ± 0.1332	<2.2 × 10^−16^	<2.2 × 10^−16^
Smoking		Missing: 1					
No(%)	762 (75.22)	28,062 (76.75)	0.2713	310 (68.89)	4656 (70.21)	0.5912	<2.2 × 10^−16^
Yes(%)	251 (24.78)	8499 (23.25)		140 (31.11)	1976 (29.79)		
Exercise		Missing: 16					
No(%)	480 (47.38)	22,981 (62.88)	<2.2 × 10^−16^	214 (47.56)	3750 (56.54)	0.0002446	<2.2 × 10^−16^
Yes(%)	533 (52.62)	13,565 (37.12)		236 (52.44)	2882 (43.46)		

^1^  *p*-values for age were calculated from Student’s *t*-tests, while the others were from chi-squared tests. ^2^ *p*-values for comparison between the mean of discovery cohort and validation cohort.

**Table 2:** Selected glaucoma-associated single nucleotide polymorphisms (SNPs) identified in Taiwan Biobank 2.0 (TWB2.0). For a complete list of all 138 genome-wide significant glaucoma-associated SNPs (*p* < 1 × 10^−5^ after SAIGE adjustments), please refer to Table S1.

SNP	CHR	Position	Nearest Gene	Minor Allele	MAF (in Cases)	MAF (in Controls)	OR	*p*-Value (SAIGE)
rs2282199	9	92147093	LINC00475	A	0.3362	0.2858	1.266	6.64 × 10^−7^
rs2282201	9	92147469	LINC00475	A	0.3362	0.2858	1.265	6.75 × 10^−7^
rs10992252	9	92150242	LINC00475	T	0.3352	0.2856	1.261	9.78 × 10^−7^
rs57413357	9	92148753	LINC00475	G	0.335	0.2857	1.259	1.11 × 10^−6^
rs59232045	9	92140079	LINC00475	A	0.3369	0.2875	1.259	1.39 × 10^−6^
rs4078356	6	24803089	RIPOR2	C	0.0447	0.0264	1.727	1.82 × 10^−6^
rs10992195	9	92009252	LINC00475	G	0.3814	0.3308	1.247	1.90 × 10^−6^
rs4757474	11	16992427	PLEKHA7	T	0.468	0.4154	1.238	2.04 × 10^−6^
rs4757472	11	16989375	PLEKHA7	G	0.4695	0.417	1.237	2.33 × 10^−6^
rs10832710	11	16991409	PLEKHA7	C	0.4685	0.417	1.236	2.48 × 10^−6^
chr11:16982833_G_GGA	11	16982833	PLEKHA7	G	0.4863	0.4333	1.238	2.49 × 10^−6^
rs4757475	11	16994869	PLEKHA7	A	0.4681	0.4159	1.236	2.53 × 10^−6^
rs10832712	11	16995533	PLEKHA7	T	0.4681	0.4159	1.236	2.53 × 10^−6^

Abbreviations: SNP, single nucleotide polymorphism; CHR, chromosome; MAF, minor allele frequency; OR, odds ratio; SAIGE, Scalable and Accurate Implementation of Generalized mixed model.

**Table 3:** Comparison of the predictive performance of Polygenic Risk Scores (PRS) with different tuning parameters, showing that the mean PRS are higher among cases than controls across all PRS models.

Tuning Parameters ^1^	Top N SNPs Included for PRS Calculation	Mean PRS		AUC [95% CI]
		Case	Control	TWB2.0
*p* ≤ 10^−5^ and r^2^ < 0.2	14	11.0835	9.8500	0.6369 [0.6194–0.6543]
*p* ≤ 10^−5^ and r^2^ < 0.4	15	12.0615	10.7101	0.6345 [0.6171–0.6519]
*p* ≤ 10^−5^ and r^2^ < 0.6	18	14.8168	13.1126	0.6105 [0.593–0.6279]
*p* ≤ 10^−5^ and r^2^ < 0.8	18	14.8185	13.1142	0.6104 [0.593–0.6278]
*p* ≤ 10^−4^ and r^2^ < 0.2	134	99.3813	89.3490	0.8387 [0.8269–0.8506]
*p* ≤ 10^−4^ and r^2^ < 0.4	138	102.1499	91.9352	0.8365 [0.8246–0.8484]
*p* ≤ 10^−4^ and r^2^ < 0.6	147	109.5890	98.3110	0.8173 [0.8043–0.8302]
*p* ≤ 10^−4^ and r^2^ < 0.8	162	124.4653	112.0043	0.7984 [0.7847–0.8121]

^1^ Tuning parameters included genome-wide significance (*p*-value) and r^2^ for LD clumping. Abbreviations: SNP, single nucleotide polymorphism; PRS, polygenic risk score; AUC [95% C.I], area under curve [95% confidence interval]; TWB2.0, Taiwan Biobank 2.0.

**Table 4:** Distribution of glaucoma cases and controls according to polygenic risk score (PRS) quantiles.

Total N = 37,575	(min, Q1]	(Q1, Q2]	(Q2, Q3]	(Q3, Q4]
Control, N = 36,562	9376	9326	9220	8640
(*n*,%)	25.64%	25.51%	25.22%	23.63%
Case, N = 1013	18	68	173	754
(*n*,%)	1.78%	6.71%	17.08%	74.43%
OR [95% C.I]	1	3.80 [2.31, 6.58]	9.77 [6.19, 16.46]	45.48 [29.38, 75.39]

Abbreviations: OR, odds ratio, with reference to the lowest PRS quantile group (min, Q1]; Q, quantile; PRS, polygenic risk score model “PRS_TWB2.0”; SNP, single nucleotide polymorphism; 95% C.I., 95% confidence interval.

**Table 5:** Risk of developing glaucoma for high groups with high polygenic risk score (PRS) group.

High PRS Group	Reference Group	OR [95% C.I.]
Top 25%	Remaining 75%	9.41 [8.17, 10.87]
Top 20%	Remaining 80%	9.72 [8.49, 11.14]
Top 10%	Remaining 90%	10.58 [9.31, 12.03]
Top 5%	Remaining 95%	13.30 [11.58, 15.26]

Abbreviations: PRS, polygenic risk score model “PRS_TWB2.0”; OR [95% C.I.], odds ratio [95% confidence interval].

## Data Availability

Publicly available data were downloaded from the following databases: The TWB genetic and phenotype datasets are available through the TWB (https://www.twbiobank.org.tw, (accessed on 28 September 2021); BBJ summary statistics: http://jenger.riken.jp/en/result, (accessed on 28 September 2021); UKBB summary statistics: http://www.nealelab.is/ukbiobank, (accessed on 28 September 2021); GWASCatlog summary statistics: https://www.ebi.ac.uk/gwas/summary-statistics (accessed on 28 September 2021).

## Supplementary Materials

The following are available online at https://www.mdpi.com/article/10.3390/jpm11111169/s1, Table S1: Lead genome-wide significant SNP for each independent locus identified in the TWB Biobank 2.0, Table S2: A number of 134 SNPs used for PRS construction.
