# Supplementary material for: Genome-Wide Polygenic Risk Score for Predicting High Risk Glaucoma Individuals of Han Chinese Ancestry
Source: J Pers Med. 2021 Nov 9;11(11):1169. doi: 10.3390/jpm11111169 (PMC8618593; doi:10.3390/jpm11111169)
Supplement: Supplementary file 1 [file jpm-11-01169-s001.zip › jpm-1419829-supplementary.pdf]

Supplementary materials

Table S1. Lead genome-wide significant SNP for each independent locus identified in the TWB Biobank 2.0.

SNP	CHR	Position	Nearest Gene	Minor allele	MAF (in cases)	MAF (in controls)	OR	p-value (SAIGE)
rs2282199	9	92147093	LINC00475	A	0.3362	0.2858	1.266	6.64×10^{-07}
rs2282201	9	92147469	LINC00475	A	0.3362	0.2858	1.265	6.75×10^{-07}
rs10992252	9	92150242	LINC00475	T	0.3352	0.2856	1.261	9.78×10^{-07}
rs57413357	9	92148753	LINC00475	G	0.335	0.2857	1.259	1.11×10^{-06}
rs59232045	9	92140079	LINC00475	A	0.3369	0.2875	1.259	1.39×10^{-06}
rs4078356	6	24803089	RIPOR2	C	0.0447	0.0264	1.727	1.82×10^{-06}
rs10992195	9	92009252	LINC00475	G	0.3814	0.3308	1.247	1.90×10^{-06}
rs4757474	11	16992427	PLEKHA7	T	0.468	0.4154	1.238	2.04×10^{-06}
rs4757472	11	16989375	PLEKHA7	G	0.4695	0.417	1.237	2.33×10^{-06}
rs10832710	11	16991409	PLEKHA7	C	0.4685	0.417	1.236	2.48×10^{-06}
chr11:16982833_G_GGA	11	16982833	PLEKHA7	G	0.4863	0.4333	1.238	2.49×10^{-06}
rs4757475	11	16994869	PLEKHA7	A	0.4681	0.4159	1.236	2.53×10^{-06}
rs10832712	11	16995533	PLEKHA7	T	0.4681	0.4159	1.236	2.53×10^{-06}
rs2302207	11	16996075	PLEKHA7	C	0.4681	0.4159	1.236	2.53×10^{-06}
rs6486334	11	16994010	PLEKHA7	T	0.4681	0.4161	1.235	2.78×10^{-06}
rs10766367	11	16991605	PLEKHA7	A	0.4681	0.4161	1.235	2.78×10^{-06}
rs10992246	9	92134082	LOC100128076	G	0.3242	0.2769	1.252	3.02×10^{-06}
rs3215938	9	92142415	LINC00475	TG	0.318	0.2718	1.249	3.70×10^{-06}
rs185821376	4	39153238	KLHL5	T	0.0303	0.0161	1.913	3.72×10^{-06}
rs6486333	11	16989125	PLEKHA7	G	0.4705	0.4192	1.231	3.82×10^{-06}
rs531682744	4	39169157	WDR19	A	0.0303	0.0161	1.909	3.95×10^{-06}
rs10832707	11	16985515	PLEKHA7	A	0.4701	0.419	1.23	4.05×10^{-06}
rs2353369	11	16985673	PLEKHA7	A	0.4701	0.419	1.23	4.05×10^{-06}
rs5789966	11	16988663	PLEKHA7	G	0.4706	0.4195	1.23	4.39×10^{-06}
rs4641129	9	92104454	SPTLC1	A	0.3426	0.2956	1.242	4.53×10^{-06}
rs11533009	9	92075550	SPTLC1	A	0.3419	0.295	1.242	4.55×10^{-06}
rs10680948	6	103579034	LOC105377916	T	0.5159	0.4646	1.228	4.69×10^{-06}
rs903990	16	76927278	LOC100128497	T	0.3068	0.3564	0.7992	4.73×10^{-06}
rs12683519	9	92126555	LOC100128076	T	0.3226	0.2763	1.247	4.90×10^{-06}
rs74658034	9	92135372	LOC100128076	G	0.3222	0.2762	1.246	4.97×10^{-06}
rs11156316	6	103582944	LOC105377916	T	0.5149	0.4637	1.228	5.03×10^{-06}
rs9377524	6	103583336	LOC105377916	T	0.5149	0.4637	1.228	5.03×10^{-06}
rs138964859	9	92109771	SPTLC1	A	0.3426	0.2958	1.241	5.05×10^{-06}
rs9386369	6	103582669	LOC105377916	G	0.5149	0.4637	1.228	5.07×10^{-06}
rs13209580	6	24802852	RIPOR2	T	0.0434	0.0261	1.696	5.11×10^{-06}
rs143676004	9	92109648	SPTLC1	T	0.3426	0.2959	1.24	5.13×10^{-06}

rs9978364	21	33017911	OLIG2	A	0.0954	0.0688	1.426	$5.13×10^{-06}$
rs10832709	11	16987097	PLEKHA7	C	0.4707	0.4202	1.227	$5.13×10^{-06}$
rs6930729	6	103575462	LOC105377916	C	0.5158	0.4649	1.226	$5.18×10^{-06}$
rs10992248	9	92134769	LOC100128076	T	0.3217	0.2757	1.246	$5.30×10^{-06}$
rs4555956	6	103569479	LOC105377916	A	0.5139	0.4628	1.227	$5.32×10^{-06}$
rs9983364	21	33014291	OLIG2	C	0.0958	0.0692	1.424	$5.39×10^{-06}$
rs34180308	21	33015073	OLIG2	A	0.0953	0.0688	1.424	$5.53×10^{-06}$
rs35997749	21	33015140	OLIG2	G	0.0953	0.0688	1.424	$5.54×10^{-06}$
rs2274921	9	92144726	LINC00475	G	0.4486	0.3987	1.227	$5.54×10^{-06}$
rs6125932	20	50183356	CEBPB-AS1	C	0.4386	0.4905	0.8113	$5.57×10^{-06}$
rs7575377	2	33302504	LTBP1	T	0.322	0.2763	1.244	$5.60×10^{-06}$
rs9322752	6	103568264	LOC105377916	C	0.5186	0.4672	1.229	$5.65×10^{-06}$
rs61584346	21	33017413	OLIG2	GAGA	0.0954	0.069	1.423	$5.67×10^{-06}$
rs139068042	9	92132811	LOC100128076	C	0.3218	0.276	1.245	$5.71×10^{-06}$
rs59856128	9	92080229	SPTLC1	A	0.3425	0.2961	1.239	$5.75×10^{-06}$
rs10992222	9	92073941	SPTLC1	T	0.3425	0.2961	1.239	$5.76×10^{-06}$
rs77450572	9	92076847	SPTLC1	T	0.3425	0.2961	1.239	$5.80×10^{-06}$
rs79161841	9	92074107	SPTLC1	A	0.3425	0.2961	1.239	$5.82×10^{-06}$
rs9399832	6	103582125	LOC105377916	G	0.5144	0.4636	1.226	$6.07×10^{-06}$
rs9386368	6	103582199	LOC105377916	T	0.5144	0.4637	1.226	$6.09×10^{-06}$
rs9386367	6	103582167	LOC105377916	G	0.5144	0.4637	1.226	$6.10×10^{-06}$
rs16856945	2	142192137	LRP1B	C	0.0351	0.02	1.786	$6.27×10^{-06}$
rs570667964	10	116602949	PNLIPRP1	T	0.0369	0.0212	1.77	$6.35×10^{-06}$
rs10465156	9	92158213	LINC00475	A	0.3312	0.2855	1.239	$6.41×10^{-06}$
rs9978551	21	33018125	OLIG2	C	0.0952	0.0689	1.422	$6.53×10^{-06}$
rs7765311	6	103569754	LOC105377916	G	0.5135	0.4627	1.225	$6.59×10^{-06}$
rs9377520	6	103577504	LOC105377916	T	0.5133	0.463	1.224	$6.60×10^{-06}$
rs6904099	6	103578097	LOC105377916	G	0.5133	0.463	1.224	$6.60×10^{-06}$
rs6928175	6	103578536	LOC105377916	A	0.5133	0.463	1.224	$6.60×10^{-06}$
rs6910768	6	103579487	LOC105377916	G	0.5133	0.463	1.224	$6.60×10^{-06}$
rs6939264	6	103579824	LOC105377916	A	0.5133	0.463	1.224	$6.60×10^{-06}$
rs9404428	6	103581089	LOC105377916	C	0.5133	0.463	1.224	$6.60×10^{-06}$
rs11156313	6	103581259	LOC105377916	C	0.5133	0.463	1.224	$6.61×10^{-06}$
rs4346882	6	103572784	LOC105377916	A	0.5133	0.463	1.223	$6.64×10^{-06}$
rs4299869	6	103572890	LOC105377916	C	0.5133	0.463	1.223	$6.64×10^{-06}$
rs4496836	6	103572939	LOC105377916	T	0.5133	0.463	1.223	$6.64×10^{-06}$
rs4352716	6	103573266	LOC105377916	A	0.5133	0.463	1.223	$6.64×10^{-06}$
rs7754349	6	103573562	LOC105377916	G	0.5133	0.463	1.223	$6.64×10^{-06}$
rs7765026	6	103574893	LOC105377916	C	0.5133	0.463	1.223	$6.64×10^{-06}$
rs6913858	6	103575156	LOC105377916	T	0.5133	0.463	1.223	$6.64×10^{-06}$

rs6571126	6	103575167	LOC105377916	G	0.5133	0.463	1.223	6.64×10^{-06}
rs6930891	6	103575535	LOC105377916	C	0.5133	0.463	1.223	6.64×10^{-06}
rs9377518	6	103576279	LOC105377916	A	0.5133	0.463	1.223	6.64×10^{-06}
rs9377519	6	103576281	LOC105377916	G	0.5133	0.463	1.223	6.64×10^{-06}
rs140885697	6	103581625	LOC105377916	TG	0.5139	0.4634	1.224	6.66×10^{-06}
rs4266523	6	103571355	LOC105377916	G	0.5134	0.4629	1.224	6.69×10^{-06}
rs4339502	6	103571485	LOC105377916	A	0.5134	0.4629	1.224	6.69×10^{-06}
rs6571122	6	103571575	LOC105377916	T	0.5134	0.4629	1.224	6.69×10^{-06}
rs6939107	6	103571691	LOC105377916	C	0.5134	0.4629	1.224	6.69×10^{-06}
rs7766897	6	103569978	LOC105377916	C	0.5134	0.4629	1.224	6.70×10^{-06}
rs10872772	6	103570221	LOC105377916	C	0.5134	0.463	1.224	6.78×10^{-06}
rs144308811	4	73976239	PF4	C	0.035	0.0199	1.786	6.81×10^{-06}
rs9391066	6	103581721	LOC105377916	T	0.5139	0.4635	1.224	6.81×10^{-06}
rs16908137	9	92081868	SPTLC1	C	0.343	0.2969	1.237	6.84×10^{-06}
rs74981514	9	92077091	SPTLC1	A	0.343	0.2969	1.237	6.86×10^{-06}
rs117063038	9	92005480	LINC00475	G	0.3477	0.3013	1.236	6.91×10^{-06}
rs139179848	9	92073635	SPTLC1	AT	0.3429	0.2967	1.237	6.91×10^{-06}
rs9391064	6	103579327	LOC105377916	G	0.5133	0.4631	1.223	7.00×10^{-06}
rs9377523	6	103580919	LOC105377916	G	0.5133	0.4632	1.222	7.21×10^{-06}
rs2096388	9	92012589	LINC00475	T	0.3484	0.302	1.235	7.32×10^{-06}
rs9391067	6	103581929	LOC105377916	C	0.5134	0.4631	1.223	7.37×10^{-06}
rs79699262	9	92011366	LINC00475	C	0.3484	0.3021	1.235	7.54×10^{-06}
rs1919102	9	91974279	LOC105376148	T	0.3982	0.3502	1.228	7.55×10^{-06}
rs1919103	9	91974307	LOC105376148	T	0.3982	0.3502	1.228	7.55×10^{-06}
rs9983084	21	33013983	OLIG2	G	0.0948	0.0688	1.418	7.57×10^{-06}
rs9391062	6	103575775	LOC105377916	C	0.5133	0.4633	1.222	7.66×10^{-06}
rs10992242	9	92130532	LOC100128076	T	0.3213	0.276	1.242	7.67×10^{-06}
rs4498413	6	103571289	LOC105377916	T	0.5134	0.4633	1.222	7.67×10^{-06}
rs7862826	9	92094537	SPTLC1	T	0.3421	0.2963	1.235	7.77×10^{-06}
rs10992230	9	92094235	SPTLC1	C	0.3421	0.2963	1.235	7.82×10^{-06}
rs143330048	9	92100041	SPTLC1	A	0.3426	0.2968	1.235	7.86×10^{-06}
rs117106774	9	91933087	ROR2	A	0.2832	0.2402	1.25	7.92×10^{-06}
rs12235310	9	92067372	SPTLC1	A	0.343	0.2972	1.235	7.95×10^{-06}
rs3858090	9	92090726	SPTLC1	G	0.3421	0.2964	1.234	7.96×10^{-06}
rs77094301	9	91933047	ROR2	A	0.2843	0.2412	1.249	8.00×10^{-06}
rs10992233	9	92113698	SPTLC1	G	0.3423	0.2965	1.235	8.24×10^{-06}
rs12235495	9	92067623	SPTLC1	A	0.343	0.2973	1.234	8.40×10^{-06}
rs16908003	9	91929362	ROR2	A	0.2826	0.2398	1.249	8.42×10^{-06}
rs76684062	9	91929345	ROR2	A	0.2826	0.2398	1.249	8.42×10^{-06}
rs115264513	9	92054849	SPTLC1	A	0.343	0.2974	1.234	8.52×10^{-06}

rs10820935	9	92047982	SPTLC1	A	0.3425	0.2969	1.234	8.55×10^{-06}
rs4365964	6	103569352	LOC105377916	T	0.5144	0.4646	1.221	8.56×10^{-06}
rs10992215	9	92053486	SPTLC1	T	0.3425	0.2969	1.234	8.63×10^{-06}
rs4365965	6	103569383	LOC105377916	T	0.5144	0.4646	1.221	8.72×10^{-06}
rs10992216	9	92053875	SPTLC1	G	0.3425	0.2969	1.234	8.79×10^{-06}
rs10992220	9	92067026	SPTLC1	A	0.343	0.2975	1.233	8.89×10^{-06}
rs59183854	9	92092344	SPTLC1	A	0.3425	0.2971	1.233	9.00×10^{-06}
rs114749238	9	92092513	SPTLC1	A	0.3425	0.2971	1.233	9.00×10^{-06}
rs138759478	6	103570522	LOC105377916	GTT	0.5149	0.4652	1.22	9.13×10^{-06}
rs62291417	3	188275909	LPP	T	0.0592	0.0876	0.6558	9.24×10^{-06}
rs58909848	9	92060845	SPTLC1	A	0.3408	0.2952	1.234	9.27×10^{-06}
rs16908135	9	92080433	SPTLC1	C	0.3411	0.2956	1.234	9.30×10^{-06}
rs7766547	6	103569761	LOC105377916	C	0.5125	0.4626	1.221	9.34×10^{-06}
rs80336008	9	92063383	SPTLC1	A	0.3337	0.2885	1.235	9.35×10^{-06}
rs145495258	3	188269977	LPP	T	0.0592	0.0876	0.656	9.44×10^{-06}
rs148651607	8	60413690	LINC01301	C	0.0266	0.0138	1.945	9.55×10^{-06}
rs11024103	11	16987261	PLEKHA7	A	0.4695	0.4203	1.221	9.56×10^{-06}
rs9391056	6	103562706	LOC105377916	G	0.5149	0.4652	1.22	9.77×10^{-06}
rs4573114	6	103567260	LOC105377916	G	0.5149	0.4652	1.22	9.78×10^{-06}
rs10457116	6	103568099	LOC105377916	T	0.5149	0.4653	1.22	9.84×10^{-06}
rs10457982	6	103568124	LOC105377916	G	0.5149	0.4653	1.22	9.84×10^{-06}
rs6571121	6	103568932	LOC105377916	A	0.5149	0.4653	1.22	9.84×10^{-06}

Abbreviations: SNP, single nucleotide polymorphism; CHR, chromosome; MAF, minor allele frequency; OR, odds ratio; SAIGE, Scalable and Accurate Implementation of Generalized mixed model.

Table S2. A number of 134 SNPs used for PRS construction.

SNP	CHR	Position	Nearest Gene	Minor allele	MAF	OR	p-value (SAIGE)
rs2282199	9	92147093	LINC00475	A	0.311	1.266	6.64×10^{-07}
rs4078356	6	24803089	RIPOR2	C	0.035525	1.727	1.82×10^{-06}
rs4757474	11	16992427	PLEKHA7	T	0.4417	1.238	2.04×10^{-06}
rs185821376	4	39153238	KLHL5	T	0.02318	1.913	3.72×10^{-06}
rs10680948	6	103579034	LOC105377916	T	0.49025	1.228	4.69×10^{-06}
rs903990	16	76927278	LOC100128497	T	0.3316	0.7992	4.73×10^{-06}
rs9978364	21	33017911	OLIG2	A	0.082095	1.426	5.13×10^{-06}
rs6125932	20	50183356	CEBPB-AS1	C	0.46455	0.8113	5.57×10^{-06}
rs7575377	2	33302504	LTBP1	T	0.29915	1.244	5.60×10^{-06}
rs16856945	2	142192137	LRP1B	C	0.027515	1.786	6.27×10^{-06}
rs570667964	10	116602949	PNLIPRP1	T	0.029035	1.77	6.35×10^{-06}
rs144308811	4	73976239	PF4	C	0.027485	1.786	6.81×10^{-06}
rs62291417	3	188275909	LPP	T	0.073415	0.6558	9.24×10^{-06}
rs148651607	8	60413690	LINC01301	C	0.02019	1.945	9.55×10^{-06}
rs62468636	7	23758453	STK31	A	0.40985	0.8125	1.01×10^{-05}
rs79887407	5	24858152	LINC02239	C	0.05816	1.492	1.12×10^{-05}
rs11042938	11	10732405	CTR9	A	0.43935	0.8198	1.20×10^{-05}
rs12042959	1	243369971	SDCCAG8	G	0.10155	1.366	1.21×10^{-05}
rs12607667	18	66710675	CDH19	T	0.057845	1.491	1.21×10^{-05}
rs7983546	13	95854973	UGGT2	G	0.3397	1.227	1.23×10^{-05}
rs2894186	6	31239091	HLA-C	C	0.37615	1.223	1.25×10^{-05}
rs117177066	8	54791167	RP1	T	0.02098	1.894	1.30×10^{-05}
rs79906218	14	87644077	LINC02330	T	0.03077	1.698	1.36×10^{-05}
rs79808547	5	135038322	PITX1-AS1	C	0.1404	1.314	1.39×10^{-05}
rs137873049	2	216947307	LOC101928278	T	0.031215	1.69	1.48×10^{-05}
rs141224943	7	113919565	PPP1R3A	T	0.041395	1.581	1.49×10^{-05}
rs7017174	8	123755573	LOC105375739	A	0.30955	0.8059	1.49×10^{-05}
rs10483616	14	52711585	PSMC6	A	0.1823	1.276	1.50×10^{-05}
rs4765771	12	4222244	LOC105369612	C	0.321	0.8096	1.68×10^{-05}
rs3765814	1	241609725	OPN3	T	0.261	0.7991	1.75×10^{-05}
rs9574198	13	78234493	OBI1-AS1	T	0.34485	1.219	1.85×10^{-05}
rs188334994	5	2370025	LOC100506858	A	0.016105	2.042	1.87×10^{-05}
rs512481	11	117848165	FXYD6	A	0.3389	1.226	1.89×10^{-05}
rs12471420	2	66421326	MEIS1-AS3	T	0.02133	1.865	1.92×10^{-05}
rs117126910	2	166678330	LOC107985958	C	0.027325	1.742	1.95×10^{-05}
rs134590	22	29070837	KREMEN1	C	0.078905	0.6767	1.95×10^{-05}
rs74425084	3	7392731	GRM7	A	0.033975	1.641	1.96×10^{-05}
rs144564223	7	6075075	USP42	C	0.01641	2.008	2.09×10^{-05}

rs144598328	3	79476725	ROBO1	C	0.025915	1.744	2.11×10^{-05}
rs183444495	4	187614498	LINC02492	C	0.024195	1.788	2.30×10^{-05}
rs79901472	2	162527527	KCNH7	A	0.02312	1.806	2.34×10^{-05}
rs11025369	11	20070850	NAV2	T	0.081985	1.389	2.37×10^{-05}
rs75421747	1	119978121	NOTCH2	A	0.021405	1.857	2.39×10^{-05}
rs12451346	17	10207255	GAS7	G	0.028055	1.711	2.46×10^{-05}
rs138275910	14	98887997	LOC105370658	T	0.15465	1.291	2.48×10^{-05}
rs35314156	1	3924464	LINC01345	G	0.43225	1.21	2.64×10^{-05}
rs2643023	17	54974641	UGGT2	T	0.3501	0.8165	2.65×10^{-05}
rs10434288	4	178628542	STXBP4	G	0.3394	1.216	2.77×10^{-05}
rs374973567	4	166732804	LOC105377563	AT	0.055185	1.471	2.80×10^{-05}
rs4960026	6	4697631	PSMC1P11	A	0.12795	1.311	2.86×10^{-05}
rs185851793	10	34841411	PARD3	A	0.017975	1.949	2.86×10^{-05}
rs9916791	17	7817913	DNAH2	T	0.4	1.212	2.90×10^{-05}
rs35016158	12	63508843	LOC105369797	T	0.09572	0.7074	2.93×10^{-05}
rs7405477	17	73854466	LINC00469	C	0.1782	1.267	2.93×10^{-05}
rs80114295	10	80325146	DYDC1	C	0.1595	1.281	3.07×10^{-05}
rs12961905	18	33626657	ASXL3	T	0.214	1.247	3.15×10^{-05}
rs1205318	19	54330126	LILRA4	T	0.14605	1.29	3.24×10^{-05}
rs76932625	16	26778944	LOC105370658	A	0.1193	1.317	3.38×10^{-05}
rs12682968	9	96599921	CDC14B	A	0.2914	1.225	3.68×10^{-05}
rs792108	2	5392660	LOC105373399	T	0.4199	1.206	3.78×10^{-05}
rs66470952	7	45050545	CCM2	T	0.1754	0.7756	4.00×10^{-05}
rs13274488	8	13111546		A	0.3585	0.8221	4.06×10^{-05}
rs192972545	11	38710626	LOC105376635	C	0.014758	2.04	4.08×10^{-05}
rs12832958	12	5053734	KCNA5	T	0.02375	1.754	4.11×10^{-05}
rs647745	6	153961984	HMGB3P19	A	0.3542	0.8223	4.13×10^{-05}
chr4:26448323_G_GA	4	26448323		G	0.42935	0.8268	4.18×10^{-05}
rs10758886	9	7581717	PPIAP33	T	0.3099	1.215	4.24×10^{-05}
rs146990800	11	120872540	GRIK4	C	0.02499	1.741	4.25×10^{-05}
rs210806	22	34125561	LINC01643	C	0.1772	0.7761	4.41×10^{-05}
rs6558101	8	29215393	HMGB1P23	G	0.02987	1.654	4.58×10^{-05}
rs10992149	9	91886397	ROR2	A	0.43805	1.2	4.65×10^{-05}
rs4362447	17	78037481	TNRC6C	T	0.46315	1.202	4.69×10^{-05}
rs10488790	11	29456790	LINC02755	A	0.159	0.7708	4.73×10^{-05}
rs127758	1	184958318	NIBAN1	A	0.35805	0.8253	4.87×10^{-05}
chr4:136027579_GA_G	4	136027579		G	0.055085	1.458	4.98×10^{-05}
rs1926854	13	22951900	LOC105370111	C	0.43045	0.8304	5.12×10^{-05}
rs10262640	7	82250748	CACNA2D1	A	0.10347	1.332	5.16×10^{-05}
rs4734150	8	107597017	PGAM1P13	G	0.10317	1.331	5.25×10^{-05}

rs201015823	3	38136761	ACAA1	C	0.019195	1.846	5.27×10^{-05}
rs66886305	16	76777764	LOC105376774	T	0.30925	1.214	5.43×10^{-05}
rs6824812	4	14965319	CPEB2-DT	A	0.05276	1.464	5.44×10^{-05}
rs117473007	9	76851185	PCA3	A	0.029525	1.645	5.44×10^{-05}
rs138996417	20	22893986	CYB5AP4	T	0.054955	1.459	5.53×10^{-05}
rs5753625	22	31458860	EIF4ENIF1	T	0.1309	1.296	5.55×10^{-05}
rs141374618	8	78206453	LOC105375911	A	0.05373	1.463	5.62×10^{-05}
rs11640687	16	58337479	GINS3	T	0.027775	1.673	5.71×10^{-05}
rs1978979	7	153321010	LOC102723686	G	0.06887	1.402	5.83×10^{-05}
rs9504807	6	696282	EXOC2	T	0.11505	1.312	5.89×10^{-05}
rs6072161	20	40842787	RNA5SP484	C	0.2338	0.8031	5.90×10^{-05}
rs145146515	9	122707539	OR1L4	T	0.032265	1.601	6.00×10^{-05}
rs34107416	5	91324306	LUCAT1	G	0.11182	1.316	6.15×10^{-05}
rs11842755	13	96130341	HS6ST3	C	0.41575	1.201	6.20×10^{-05}
rs75039125	9	76970482	LYPLA2P3	A	0.02875	1.654	6.25×10^{-05}
chr3:68499264_G_GA	3	68499264	TAFA1	GA	0.14545	0.7666	6.34×10^{-05}
rs74387450	3	74801318	LOC105377167	A	0.017765	1.888	6.35×10^{-05}
rs675431	5	31352601	CDH6	C	0.44085	1.197	6.46×10^{-05}
rs197344	7	36379204	KIAA0895	T	0.086755	0.7088	6.51×10^{-05}
rs796198509	14	32214341	AKAP6	A	0.016735	1.915	6.52×10^{-05}
rs146872424	5	162178858	GABRG2	A	0.031065	1.628	6.56×10^{-05}
rs138329777	9	4262041	GLIS3	G	0.01971	1.823	6.90×10^{-05}
chr9:109114076_CCAAAA_C	9	109114076	TMEM245	C	0.106285	0.7336	6.91×10^{-05}
rs138563247	6	40080665	TUBBP9	T	0.014965	1.974	6.92×10^{-05}
rs117436899	19	2923745	LOC101928631	A	0.02653	1.676	6.92×10^{-05}
rs5771285	22	50257479	MAPK12	G	0.14425	0.7653	7.13×10^{-05}
rs12136516	1	103592647	AMY2B	C	0.1349	1.299	7.19×10^{-05}
rs78350134	11	69307450	IFITM9P	A	0.11204	1.312	7.21×10^{-05}
rs6599389	4	945325	TMEM175	A	0.2951	0.8193	7.29×10^{-05}
rs149162405	14	77610068	SPTLC2	T	0.02006	1.796	7.43×10^{-05}
rs2024556	20	2704563	EBF4	T	0.45665	0.8345	7.70×10^{-05}
rs142497682	18	66082449	PRPF19P1	T	0.034055	1.58	7.71×10^{-05}
rs199515533	16	68981750	TANGO6	T	0.01573	1.946	7.81×10^{-05}
rs140046717	11	38383498	LOC105376634	A	0.02102	1.772	7.91×10^{-05}
rs143727079	5	152650215	LINC01470	T	0.017755	1.864	7.98×10^{-05}
rs148140115	2	201592870	C2CD6	C	0.32695	1.204	8.13×10^{-05}
rs61168622	18	59968631	NFE2L3P1	C	0.04837	1.474	8.15×10^{-05}
rs2814744	9	102059384	ARL2BPP7	A	0.08114	1.364	8.28×10^{-05}
rs373614297	3	31100809	CNN2P6	A	0.020705	1.789	8.32×10^{-05}
rs2846054	11	123435258	GRAMD1B	T	0.20665	1.238	8.55×10^{-05}

rs7653174	3	159120808	IQCJ	G	0.4557	0.8376	8.56×10^{-05}
rs73338489	14	42743295	YWHAQP1	T	0.1214	1.295	8.65×10^{-05}
rs9682469	3	168696909	EGFEM1P	C	0.1304	1.289	8.74×10^{-05}
rs117361458	22	38523488	DMC1	C	0.02931	1.63	8.79×10^{-05}
rs117968369	6	138755564	CCDC28A-AS1	A	0.037235	1.544	8.80×10^{-05}
rs9263993	6	31224989	PSORS1C3	T	0.21175	0.8003	8.90×10^{-05}
rs142926063	11	80308264	LOC105369406	A	0.02643	0.5216	8.93×10^{-05}
rs183957928	9	114104963	KIF12	T	0.019225	1.812	8.98×10^{-05}
rs59458224	14	70459856	ADAM21	C	0.3173	1.205	9.01×10^{-05}
rs1855617	1	244338681	LOC105373262	G	0.3588	1.2	9.11×10^{-05}
rs117859593	15	48107072	SLC24A5	G	0.02187	1.738	9.19×10^{-05}
rs79114593	3	4529455	ITPR1	A	0.03862	0.5933	9.37×10^{-05}
rs78927261	11	76518745	EMSY	A	0.02303	1.731	9.44×10^{-05}
rs144663554	4	10244048	RAF1P1	C	0.018005	1.855	9.49×10^{-05}
rs9840873	3	90001814	PROS2P	A	0.07785	1.366	9.92×10^{-05}
rs1943716	11	84853763	DLG2	C	0.39945	0.8369	9.93×10^{-05}

Abbreviations: SNP, single nucleotide polymorphism; CHR, chromosome; MAF, minor allele frequency; OR, odds ratio; SAIGE, Scalable and Accurate Implementation of Generalized mixed model.
